# Planning National Radiotherapy Services

**DOI:** 10.3389/fonc.2014.00315

**Published:** 2014-11-25

**Authors:** Eduardo Rosenblatt

**Affiliations:** ^1^Applied Radiation Biology and Radiotherapy Section, Division of Human Health, International Atomic Energy Agency, Vienna, Austria

**Keywords:** planning, national, radiotherapy, services, cancer, treatment

## Abstract

Countries, states, and island nations often need forward planning of their radiotherapy services driven by different motives. Countries without radiotherapy services sponsor patients to receive radiotherapy abroad. They often engage professionals for a feasibility study in order to establish whether it would be more cost-beneficial to establish a radiotherapy facility. Countries where radiotherapy services have developed without any central planning, find themselves in situations where many of the available centers are private and thus inaccessible for a majority of patients with limited resources. Government may decide to plan ahead when a significant exodus of cancer patients travel to another country for treatment, thus exposing the failure of the country to provide this medical service for its citizens. In developed countries, the trigger has been the existence of highly visible waiting lists for radiotherapy revealing a shortage of radiotherapy equipment. This paper suggests that there should be a systematic and comprehensive process of long-term planning of radiotherapy services at the national level, taking into account the regulatory infrastructure for radiation protection, planning of centers, equipment, staff, education programs, quality assurance, and sustainability aspects. Realistic budgetary and cost considerations must also be part of the project proposal or business plan.

## Introduction

The contribution of radiotherapy to cancer treatment is significant. Radiotherapy represents one of the three pillars of cancer treatment (with surgery and systemic therapies) and in multiple studies has proven to be a cost-effective modality for cure and palliation. The impact of radiotherapy in cancer cure has been estimated at 40%, compared to 49% of patients being cured by surgery, and 11% of patients by systemic treatments (1).

## Objective and Context

The objective of radiotherapy services is the delivery of an adequate radiotherapy treatment to all patients who need it, within a culture of safety awareness. The optimal yield of radiotherapy services occurs when they are integrated into effective healthcare systems and functional national cancer control plans. The reason is simple. In countries without a coordinated national cancer control plan, cancer patients are treated when they are diagnosed, often presenting in advanced stages of disease. This in turn determines that the majority of patients are treated for palliation.

**Figure 1:**
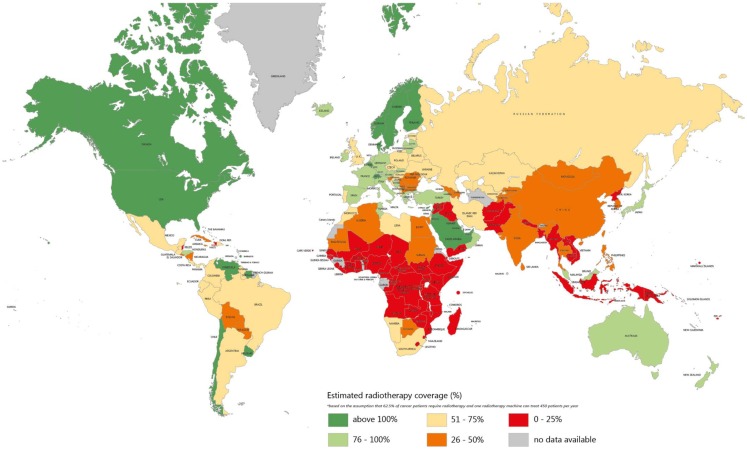
**Estimation of radiotherapy needs coverage worldwide**. The colors indicate the estimated coverage of the need by existing radiotherapy equipment. Source of data: Refs. (24) and (25).

In countries or states with an effective national cancer control plan that includes preventive, early detection, and screening programs, an increased number of patients are diagnosed at an early disease stage, treated effectively, and therefore the treatment outcomes of radiotherapy improve (Figure 1).

## Safety First

Government plays a central role in the establishment of normative and regulation of the use of radiation in medicine, which needs to be satisfied before introducing radiotherapy into a country. Meeting the regulatory requirements will go toward satisfying the radiation protection and safety aspects of establishing radiotherapy services. The range of regulatory requirements varies from country to country, but the IAEA has established, through the provision of safety standards (2), the essential components of a required regulatory infrastructure for radiation protection and safety. Regulations for the use of ionizing radiation in medicine are established in respect of the governmental, legal and regulatory framework for safety. The objective is to protect the public health and safety by preventing the availability of unsafe practices and equipment. Radiation exposure of human beings should only be considered when it is effective and potentially beneficial for diagnosis or treatment. Needless or excessive exposures are not justified and patients should be guaranteed that the treatment is reliable and that individuals administering radiotherapy are adequately trained.

Safety is the primary regulatory goal. Excessive or non-existing regulations can prohibit access to radiotherapy. A country’s regulatory infrastructure needs to be in place in order to balance safety, effectiveness, the need for medical radiation practices, and access to therapy. Regulations must be in place to facilitate informed and rational decision-making and to protect against unwise, ill-informed, or negligent practices.

Dunscombe (3) made an analysis of seven sources of radiotherapy safety recommendations and distilled from them the 12 most frequently recommended initiatives. The 12 recommended initiatives were: (1) staff training, (2) adequate staffing levels, (3) adequate documentation/standard operating procedures, (4) voluntary incident learning system, (5) quality communication, (6) use of check lists, (7) quality control and preventive maintenance, (8) dosimetric audits, (9) radiation oncology specific accreditation, (10) minimizing interruptions, (11) prospective risk assessment, and (12) a safety culture.

## Estimating Demand

How many teletherapy machines should be operational in a country in order to completely cover demand? This is a challenging question since there are large variations in radiotherapy utilization (RTU) among countries. In this discussion, we use the term teletherapy machine to refer to all including cobalt-60 units, medical accelerators, helical tomotherapy devices, and robotic radiotherapy. RTU benchmarks can be derived from evidence-based guidelines, criterion based, or based on a retrospective examination of actual practice.

Estimating demand means knowing how many *patients* will require a radiotherapy course in any given year or better yet how many *courses* of radiotherapy will be given since some patients may require more than one course. A more refined method consists in estimating the number of fractions that will be applied based on the cancer spectrum of diseases and stages. The number of new cancer cases per year in a given population (crude incidence) can be obtained from a national population based cancer registry in countries that have a reliable operational one. For countries where this variable is not measured, the International Agency for the Research on Cancer (IARC) provides a best estimate of crude incidence, which is reflected in their database Globocan-2012 (8).

Only a fraction of all cancer patients will require radiotherapy, which leads to the concept of RTU rate. Approximately, 48–62% of all cancer patients’ benefit from radiation therapy (9–11). This depends on the extent of disease at presentation and the profiles of cancer observed in a specific population. A RTU of 50% would then be a good approximation to this value for developed and middle-income countries. There is no evidence-based data for low-income countries. The total number of teletherapy machines required in a given country is given by the total number of “radiotherapy courses” in a year, divided by the teletherapy machine use. The teletherapy machine use is the number of radiotherapy courses delivered by one teletherapy machine in 1 year. The ESTRO/QUARTS Project (12) estimated a teletherapy machine use of 450 courses/year at that time. This benchmark is questioned today since the radiotherapy practice has changed significantly with the introduction of new technologies and fractionation schedules. However, an alternate benchmark that reflects current practice has not been determined so far. A more sophisticated approach to demand calculation can be attempted taking into account the full spectrum of diseases and their stages in a particular country, and the proportion of patients that will require IMRT techniques and its variations as opposed to 2D or 3D conformal techniques.

Data from Australia (13) indicates that a curative course of radiotherapy requires an average of 22 fractions and a palliative course four fractions, thus the total average would be 18 fractions per first course. The average linear accelerator treats four to five patients per hour, so the total linac utilization will depend on the total number of hours per day that the machine is active. In Canada, the average time of machine operation per day is 10 h.

## Radiotherapy Centers

A radiotherapy center is a medical department where patients are treated with usually megavoltage radiotherapy. The definition is not redundant. Centers that use orthovoltage only for skin lesions, radiosurgery only for intracranial disease, brachytherapy only or radiotherapy for veterinarian applications are not considered radiotherapy centers. International regulations on safety require that treatments with ionizing radiation be prescribed by a physician trained and licensed in this discipline and the dosimetry monitored by a trained medical physicist.

Radiotherapy centers location should follow the population concentration distribution in a country. A single center may suffice in small countries or even in large countries with a small population if transport services between population centers are adequate. The centralized comprehensive facility model may be adequate when the distances involved are short, but for longer distances, a fully decentralized service is warranted (14).

In large countries, a network of oncology services will be required, with a radiotherapy center within each region. For those patients, living at a distance from the radiotherapy center, funding will have to be set aside to cover for costs of transport and accommodation facilities, in particular for pediatric patients and their families. Countries where a significant proportion of the population are living at a distance or geographically isolated from the main centers, may also consider either the implementation of consultation clinics as focal points for further referral (primary care clinics can fulfill this role) or alternatively facilitate patient commuting through an organized transport service.

A study from Ontario (11) showed that the province’s highly centralized radiotherapy network did not provide adequate or equitable access to care to the province’s dispersed population. In this study, the actual RTU rate was 29%, which is lower than the generally accepted rate for a developed country. A similar study from the North of England showed socio-economic gradients in access to services (15) related to education levels and car use.

A radiotherapy center or department should be specifically planned and designed to fulfill its role, in terms of appropriate patient flow, location of the treatment machines, waiting rooms, physicians’ offices, and patient examination rooms, planning rooms, mold room, storage, and others as required.

Once the decision to establish a radiotherapy facility has been made, careful co-ordination, and monitoring of the planning and timelines is key to the project’s success. The professional team required to design, construct, and commission a radiotherapy facility needs to be multi-disciplinary because the project not only involves the construction of specialized bunkers to house the radiotherapy imaging and treatment equipment but also needs to take into account the clinical workflow as well as anticipate non-disruptive expansion in the future. Since the process of radiotherapy is closely related to key staff functions, the detail of the internal design of the facility is important to achieving sound work-place ergonomics and to facilitate workflow. An overall concept design should therefore consist of the five key functional areas, which expedite radiotherapy workflow. These functional areas are the reception, clinical consulting areas, the imaging and treatment planning area, and the treatment suites (teletherapy and brachytherapy). The relative placement of these areas should be adapted to the proposed site and preferred local practice; however, it should expedite broader staff and patient movement, consultation, and communication. The position of the major equipment at the various duty stations within each functional area is provided for in “International Atomic Energy Agency (IAEA) Radiotherapy facilities; master planning and concept design considerations (16)”. Expansion route possibilities are also indicated.

Clinically qualified medical physicists are responsible for ensuring that the shielding calculations are based on acceptable estimates of the projected local workload, use, and occupancy factors, and that the design accommodates the desired clinical workflow. In addition, the future implementation of new techniques and technologies should also be considered. The national radiation safety regulator is mandated to approve the final design prior to construction, and license the facility prior to the initiation of operations. Timeline synchronization between building a radiotherapy facility, procurement, and installation of equipment and training of staff is very important and has to be planned carefully. If the equipment is installed but the team has not completed their training, the result will be a non-operational facility, which is generating costs but not treating patients. Conversely, if staff completes their training long before the facility is ready, members may be compelled to take other job positions, change career, or emigrate in search of their livelihood. Our experience indicates that training of a radiotherapy team should start roughly 2 years before the initiation of construction. Funds for staff training must be allocated early and be part of the initial business plan or project proposal.

## Equipment

A basic radiotherapy center aiming at treating an average of 1000 patients/year should be equipped with at least a single-photon energy teletherapy unit, an orthovoltage unit, a brachytherapy afterloader (ideally for high dose-rate brachytherapy), an X-ray C-arm, full range of applicators, a simulator, preferably a CT-simulator, a computerized treatment planning system (TPS), film processing equipment, patient immobilization devices, and mold room equipment, beam measurement and quality assurance (QA) equipment. A second teletherapy unit may become necessary to expedite workflow and for back-up.

Procurement of new equipment has to be implemented through a transparent tendering process. Since technological developments in radiotherapy occur much faster than the economic lifetime of a linear accelerator, larger radiotherapy centers, which replace one or more machines every few years, enable the introduction of new technology at a faster rate.

The cost and cost-benefit of radiotherapy has been extensively studied. The cost of radiotherapy in a given facility tends to rise as the number of treated patients decreases below 1600, and extended hours of operation do not appear to generate significant, if any, savings when realistic assumptions about machine lifetime and overtime payments are made (17).

## Staffing and Education

A very important consideration is staffing levels. There is very little evidence-based documentation that precisely quantifies the number and type of professionals needed to support a service that is also directly related to patient workload, technology, techniques, procedures, and infrastructure. As a result, initiation of new radiotherapy services in low and middle-income countries has traditionally been planned in accordance with IAEA guidelines, which list a suite of equipment constituting a basic service that is resourced by a core number of professionals who attend to a given patient workload (18, 19). These professionals, including radiation oncologists and medical physicists, are required in the practice of radiotherapy under the IAEA International Basic Safety Standards (2).

The aforementioned basic department should have four to five radiation oncologists, three to four medical physicists, seven RTTs, three radiotherapy nurses, and one maintenance technician/engineer. Staff numbers and training should be adapted to the number of patients treated, the case-mix, the number of courses given per year, the activities performed and the level of complexity of the equipment and techniques. Staffing requirements vary greatly depending on case-mix, type, and complexity of the techniques, research, and teaching commitments. Given the complexities of today’s modern radiotherapy clinics, rather than give fixed recommendations for staff numbers, the current approach is to use an algorithm that will provide the number of staffs needed for a department according to the activities implemented.

Staffing levels in the clinical environment are not only important for planning and budgetary purposes and fundamental to quality patient care and safety but they are often also specified for practice accreditation purposes and professional credentialing. The estimation of reasonable staffing levels to support radiotherapy services has often been loosely based on patient population size, infrastructure, equipment availability, and disease incidence. Retrospective subjective estimates based on existing practice are often the benchmark for predicting future staffing needs locally. Detailed measurements of how long each procedure or activity takes to perform is probably the most objective basic evidence required to estimate full-time equivalent staffing levels (20). Such measurements are logically more useful and valid if they are performed in a variety of clinics, for a range of services and applied to professionals with a wide range of experience.

## Access

The concept of access (or accessibility) to radiotherapy services refers to the fact that these medical services can be utilized by all patients who need them. Access includes availability, accessibility, affordability, accommodation, and awareness of health professionals and the public. The existence of radiotherapy departments or services in a country (availability) is a necessary but not sufficient condition for access. For example, a clinic may be geographically inaccessible to patients residing in another region of the country. Or a majority of available clinics in a given country may be private clinics demanding payment for service, which makes them inaccessible to a significant sector of the population below the poverty level. It is the government’s responsibility through its ministry of health to ensure access to radiotherapy services to all cancer patients who need them.

## Quality and Sustainability

Quality in radiotherapy means providing a service that satisfies patient’s expectations follows optimal professional practice by obtaining optimal results and fulfils the regulatory requirements at a minimal cost and without waste of resources. Thus, quality in radiotherapy has different meanings from the perspective of the patient, the professional, or the administrator.

The concept of total quality management (TQM) consists of organization-wide efforts to install and make permanent a climate in which an organization continuously improves its ability to deliver high-quality products or services to customers, has been borrowed from the industry, particularly from the standardized approach to quality called ISO (21). It is a set of control points that ensures that each element of a process or a series of processes conforms to a pre-established standard. The idea behind it is that if a process conforms to its standards, then the result will actually meet the expectations. In radiotherapy, the expectations are the control of a cancer with minimal and predictable negative impact on quality-of-life.

Quality can be assessed by three different approaches (22): by the infrastructure, processes, or outcomes.

Infrastructure: the rationale is that quality can only be produced within an appropriate infrastructure (buildings, staffing, competences and equipment). Process: a second approach is process control. It is based on the observation that if a process conforms to a standard, then the quality of its results is predictable. Outcomes: the ultimate goal of radiotherapy, as mentioned earlier, is disease control. Five-year survival, years of survival adjusted for quality-of-life (“quality-adjusted life years”; QUALY), local control, and other clinical endpoints are all legitimate measurements of the appropriateness of radiotherapy interventions.

To assess quality in countries with established services, it is recommended to conduct an annual survey of production, equipment, and personnel of radiotherapy centers. This should include questions on the number and type of external beam and brachytherapy treatment equipment, absolute number, and number of full-time equivalent radiation oncologists, medical physicists, and radiation technologists and support personnel number of persons in training and vacancies. It is also advisable to select a set of validated quality indicators and apply this set year after year to document the dynamics of the radiotherapy system as a whole.

Budgetary provisions must be set aside for the maintenance of equipment, maintenance service and repairs, replacement of parts and sources, overheads and consumables and training and education of staff.

Radiotherapy services should be patient-centered. This means that the facilities should offer convenience for the patients and families, and patient’s priorities and needs are respected. Main aspects of the service that are important to patients include: receiving the highest level of medical care, a reduction of the waiting time between diagnosis and treatment, appropriate communication with medical and other healthcare staff, obtaining information about their condition and its treatment and convenience of access.

Avoiding excessive waiting time (more than 14 days) and waiting lists is particularly important. Excessive waiting time for radiotherapy increases the risk of local tumor recurrence and eventual treatment failure (23). Waiting lists for radiotherapy are a highly visible indicator of the inability of the healthcare system to provide the service needed. Patients and families are understandably very sensitive to this problem. They may approach the media. In several countries, the direct intervention of government even through specific normative has resulted in the reduction or elimination of waiting lists.

## Conclusion

Obstacles to the effectiveness and efficiency of radiotherapy services at country level include: (1) the lack of a network type organizational structure that would link radiotherapy centers in such a way that it ensures access to a wide range of radiotherapy techniques available, (2) a limited quality management culture with services oriented to the professionals more than to the patients, (3) work organization oriented to the day-to-day practice rather than a medium or long-term strategic planning, and (4) lack of a system of self-evaluation based on carefully recorded clinical outcomes.

Observation and analysis of radiotherapy services planning around the world show that the optimal provision and outcomes are reached when (1) radiotherapy services are centrally planned and monitored through the continued use of validated indicators over time, (2) radiotherapy services are integrated into national cancer control plans, (3) local problems of access to radiotherapy services are systematically identified and addressed, (4) radiotherapy services are given the necessary attention through a combination of political will tapping into resources from government, international organizations and NGOs.

## Conflict of Interest Statement

The author declares that the research was conducted in the absence of any commercial or financial relationships that could be construed as a potential conflict of interest.

## References

[1] SBU – The Swedish Council on Technology Assessment in Health Care. Radiotherapy for cancer. Acta Oncol (1996) 35(Suppl 6):1–26.

[2] International Atomic Energy Agency. Radiation Protection and Safety of Radiation Sources: International Basic Safety Standards (Interim Edition), General Safety Requirements Part 3 No. GSR Part 3 (Interim). Vienna: IAEA (2011).

[3] Dunscombe P. Recommendations for safer radiotherapy: what’s the message? Front Oncol (2012) 2:129. 10.3389/fonc.2012.00129 23061045 PMC3460278

[4] The Royal Australian New Zealand College of Radiology. Tripartite National Strategic Plan for Radiation Oncology 2012–2020. Sydney: Royal Australian New Zealand College of Radiology (2012).

[5] Expert Working Group on the Development of Radiotherapy Services. The Development of Radiation Oncology Services in Ireland. (2003). Available from: http://www.hse.ie/eng/services/list/5/nccp/pubs/reports/Development_of_Radiation_Oncology_in_Ireland.pdf

[6] Slotman BJ Vos PH. Planning of radiotherapy capacity and productivity. Radiother Oncol (2013) 106:266–70. 10.1016/j.radonc.2013.02.006 23474286

[7] Erridgea SC Featherstone C Chalmers R Campbell J Stockton D Black R. What will be the radiotherapy machine capacity required for optimal delivery of radiotherapy in Scotland in 2015? Eur J Cancer (2007) 43:1802–9. 17616389 10.1016/j.ejca.2007.05.022

[8] International Agency for the Research on Cancer (IARC) Globocan-2012. Available from: http://globocan.iarc.fr/Default.aspx

[9] Delaney G Jacob S Featherstone C Barton M. The role of radiotherapy in cancer treatment: estimating optimal utilization from a review of evidence-based clinical guidelines. Cancer (2005) 104(6):1129–37. 10.1002/cncr.21324 16080176

[10] Barton MB Jacob S Shafiq J Wong K Thompson SR Hanna TP Estimating the demand for radiotherapy from the evidence: a review of changes from 2003 to 2012. Radiother Oncol (2014) 112(1):140–4. 10.1016/j.radonc.2014.03.024 24833561

[11] Mackillop WJ Zhou S Groome P Dixon P Cummings BJ Hayter C Changes in the use of radiotherapy in Ontario 1984–1995. Int J Radiat Oncol Biol Phys (1999) 44(2):355–62. 10760431 10.1016/s0360-3016(99)00010-3

[12] Bentzen SM Heeren G Cottier B Slotman B Glimelius B Lievens Y Towards evidence-based guidelines for radiotherapy infrastructure and staffing needs in Europe: the ESTRO QUARTS project. Radiother Oncol (2005) 75(3):355–65. 10.1016/j.radonc.2004.12.007 16086915

[13] Review of Optimal Radiotherapy Utilisation Rates. Australia: Ingham Institute for Applied Medical Research (IIAMR) – Collaboration for Cancer Outcomes Research and Evaluation (CCORE) (2013).

[14] Dunscombe P Roberts G. Radiotherapy service delivery models for a dispersed patient population. Clin Oncol (2001) 13(1):29–37. 10.1053/clon.2001.9211 11292133

[15] Jones AP Haynes R Sauerzapf V Crawford SM Zhao H Forman D. Travel time to hospital and treatment for breast, colon, rectum, lung, ovary and prostate cancer. Eur J Cancer (2008) 44:992–9. 10.1016/j.ejca.2008.02.001 18375117

[16] International Atomic Energy Agency (IAEA). Radiotherapy Facilities: Master Planning and Concept Design Considerations. Vienna: IAEA, Human Health Reports 10 (2014).

[17] Dunscombe P Roberts G Walker J. The cost of radiotherapy as a function of facility size and hours of operation. Br J Radiol (1999) 72(858):598–603. 10.1259/bjr.72.858.10560343 10560343

[18] International Atomic Energy Agency (IAEA). Setting up a Radiotherapy Programme: Clinical, Medical Physics, Radiation Protection and Safety Aspects. (2008).

[19] International Atomic Energy Agency. Planning National Radiotherapy Services: A Practical Tool. Vienna: IAEA (2010).

[20] American Society of Radiation Oncology. Safety is No Accident: A Framework for Quality Radiation Oncology and Care. ASTRO and other societies (2012).

[21] Tsim YC Yeung VWS Leung ETC An adaptation to ISO 9001:2000 for certified organisations. Manag Audit J (2002) 17(5):245 10.1108/02686900210429669

[22] Donabedian A Evaluating the quality of medical care. Milbank Quarterly (2005) 83(4):691–729 10.1111/j.1468-0009.2005.00397.x 16279964 PMC2690293

[23] Chen Z King W Pearcey R Kerba M Mackillop WJ. The relationship between waiting time for radiotherapy and clinical outcomes: a systematic review of the literature. Radiother Oncol (2008) 87(1):3–16. 18160158 10.1016/j.radonc.2007.11.016

[24] Ferlay J Soerjomatsram I Ervik M Dikshit R Eser S Mathers C GLOBOCAN 2012v1.0, Cancer Incidence and Mortality Worldwide: IARC Cancerbase No.11 [Internet]. Lyon, France: International agency for research on cancer (2013). Available from: http://globocan.iarc.fr

[25] International Atomic Energy Agency (IAEA). Directory of Radiotherapy Centres (DIRAC). (2014). Available from: http://www-naweb.iaea.org/nahu/dirac/default.asp

